# The perimenopause, depressive disorders, and hormonal variability

**DOI:** 10.1590/S1516-31802001000200008

**Published:** 2001-03-02

**Authors:** Cláudio de Novaes Soares, Lee Stuart Cohen

**Keywords:** Perimenopause, Depression, Review, Estrogen, Treatment, Peri-menopausa, Depressão, Revisão, Estrógenos, Tratamento

## Abstract

**CONTEXT:**

Several investigations have postulated that the perimenopause may represent a period of increased psychiatric vulnerability, particularly for mood disorders. This review characterizes the perimenopause, including biological changes, the influence of psychosocial factors and the most common clinical manifestations. Clinic-based studies and community-based surveys addressing the prevalence of depressive symptoms in perimenopausal women are critically reviewed. We also discuss the potential greater vulnerability to mood disturbance during the perimenopause in response to hormonal variability. A therapeutic algorithm for management of depressive symptoms in middle-aged perimenopausal women is also presented. The role of estrogen in the treatment of perimenopausal depressive symptoms is particularly discussed. In addition, we review the existing data regarding the potential efficacy of estrogen as an antidepressant agent (monotherapy, augmentation strategy or prophylaxis).

**DESIGN:**

Narrative review.

## RESUMO

**CONTEXTO:**

Diversos trabalhos têm apontado a peri-menopausa como um período de maior vulnerabilidade para o surgimento de transtornos psiquiátricos, particularmente os quadros depressivos. Esse artigo revisa, primeiramente, o conceito de peri-menopausa, descrevendo suas características clínicas, alterações biológicas e aspectos psicossociais. Estudos sobre a prevalência de transtornos depressivos durante a peri-menopausa, abordando diferentes sub-populações (mulheres atendidas em serviços ginecológicos especializados, estudos populacionais transversais e longitudinais), são discutidos de forma crítica. Nesse artigo também abordamos as alterações hormonais presentes durante a transição menopausal, e o seu possível papel de "gatilho" no surgimento/exacerbação de quadros depressivos. Apresentamos um modelo de abordagem terapêutica para os quadros depressivos surgidos entre mulheres na faixa etária correspondente à peri-menopausa. O papel da terapêutica de reposição hormonal, nesses quadros, é discutido através da revisão dos estudos existentes sobre o uso de estrógenos como agente antidepressivo (monoterapia, terapêutica adjuvante ou profilaxia).

**TIPO DE ESTUDO:**

Revisão narrativa

## Perimenopause: biology ○○and psychosocial ○aspects

### Clinical characteristics and hormonal profile

The World Health Organization defines perimenopause as the period (2-8 years) preceding menopause and the one-year period after final menses, resulting from the loss of ovarian follicular activity.^1,2^ The initiation of perimenopause is characterized by intense biological variability secondary to endocrinological and clinical changes.^3,4^ Thus, the perimenopause or so-called "menopausal transition" represents the passage from reproductive to non-reproductive life.

The perimenopause usually begins at a mean age of 45.5-47.5 years^5^ and has an average duration of 4 years until menopause occurs^6^ (mean age of 51.3 years). It is possible that the timing of menopause may be influenced by different factors such as cigarette smoking, living at high altitudes and history of depression.^7-9^ The majority of perimenopausal women experience irregular menstrual periods, when shortened cycles or longer periods of amenorrhea may reflect the large fluctuation of the ovarian estrogen secretion observed during this time. Generally, the first apparent hormonal change of the perimenopause is a rising concentration of the pituitary gonadotrophin follicle-stimulating hormone (FSH). The rising FSH concentration is probably caused by an exponential decline of gonadotrophin-sensitive ovarian follicles as menopause approaches.

Follicular development at this time has been demonstrated to be erratic, with consequent variability in estrogen levels and an increased percentage of anovulatory cycles.^10^ Thus, the pituitary gland is stimulated to produce more FSH in an effort to stimulate the resistant follicles. The production of FSH and luteinizing hormone (LH) by the pituitary gland are subject to a predominantly negative feedback by the ovarian sex steroids (estrogen and progesterone). During the menopausal transition, there may be a fluctuation of circulating levels of sex steroids, particularly a reduction of estrogens and inhibin – the latter produced in the granular cells of the ovary. This fluctuation may disturb the negative feedback of the pituitary FSH secretion, while LH production may remain in the normal range.^2^ However, serum levels of FSH and estrogens (estradiol, estrone) can widely fluctuate from cycle to cycle and from woman to woman during the perimenopause. Consequently, confirmation of the perimenopause is usually based on laboratory tests and a woman's medical history, as well as on the characteristics of somatic and emotional symptoms she reports.^3,4^ After one year of amenorrhea (postmenopause), women experience a well-defined profile of hormones, with low estrogen and progesterone, and high gonadotrophin levels.

In addition to changes in the hormonal profile, the perimenopause is typically marked by the presence of vasomotor symptoms such as hot flushes and night sweats (hot flushes that occur with perspiration during sleep). These symptoms are experienced by 45-85% of women.^11,12^ Hot flushes are similar to a heat dissipation response,^13^ and their complete physiological mechanisms remain undetermined. One hypothesis regarding the mechanism of hot flushes suggests that the changes observed in the thermoregulatory system in the hypothalamus, possibly modulated by fluctuations in levels of sex steroids and the opioid peptides, play an important role.^14^ Thus, hot flushes would be more likely to occur in response to hormonal fluctuations rather than simply to the decline of levels of estrogen.^2^ Nonetheless, some clinical studies suggest that estrogen administration decreases the occurrence of hot flushes in a dose-dependent fash-ion.^15,16^ In addition, a community-based longitudinal study investigating 453 pre-, peri-and postmenopausal women (aged 48-59 years), has also demonstrated that the frequency of hot flushes correlates with serum levels of FSH and estradiol in perimenopausal women.^17^

Physiological changes and some physical manifestations associated with the transition to menopause include urogenital atrophy causing dyspareunia, dysfunctional uterine bleeding, and a higher risk for osteoporosis and cardiovascular disease. These changes may account for the high frequency with which perimenopausal women seek medical consultation as compared to pre- or postmenopausal women.^18^

The role of socio-cultural factors and demographic characteristics Several studies have tried to correlate wellbeing during the perimenopause with different demographic characteristics and other conditions, including socioeconomic status, ethnicity, marital satisfaction and quality of family relationships.^19,20^ Conventionally, the menopausal transition has been identified as a non-adaptive event,^21^ during which women are at risk of losing a "major role," i.e. maternity. Thus, the "empty-nest syndrome" (when children leave home) has been proposed as a psychosocial cause of psychological symptoms manifesting during the menopausal transition. The relative validity of this theory appears to be restricted to women who are too engaged and over-involved with their children, and would consequently feel useless, isolated and depressed when the children leave home.^22^ Conversely, more psychologically healthy women would consider this period an opportunity to return to work and to dedicate more time to leisure activities and the marital relationship.^23^

Studies of Japanese society conducted and reviewed by Lock^24,25^ suggest that the experience of menopause may vary considerably. Compared to North American women, Japanese women experienced fewer menopausal somatic and emotional symptoms (i.e., depression, anxiety, night sweats and particularly hot flushes, the latter considered a "true" symptom of menopause), and a lower rate of osteoporosis, breast cancer and cardiovascular disease. Lock postulates that these findings could be correlated with different cultural and biological factors, such as the relatively higher value of older women in Japanese culture, as well as the fact that the Asian diet contains many soy products, which may influence hormonal profiles in peri/postmenopausal women. A recent survey including more than 4000 Japanese women (aged 45-55) also confirmed the association between dietary factors and the timing of menopause. High calcium and soy product intakes were significantly associated with menopause at a later age (ORs = 1.25 and 1.42, respectively). Conversely, higher intakes of fat, cholesterol, and coffee – more frequently observed in occidental societies - were inversely and significantly associated with later menopause (ORs for the highest thirds of fat, cholesterol and coffee intakes were 0.78, 0.79, and 0.70, respectively).^26^

The degree to which demographic characteristics and habits may influence the menopausal transition depends on the population investigated. Studies based on women attending menopause clinics ^27-29^ note high rates of physical (up to 96%) and emotional complaints (up to 63%), which are poorly correlated with marital status, employment or lifestyle. A cross-sectional community-based study in England (pre-, peri- and postmenopausal women) suggested, on the other hand, that the presence of somatic symptoms, depressed mood and sleep problems could be predicted by social class and absence of employment. In addition, a recent study of more than 500 pre, peri- and postmenopausal women, in a primary-care clinic in Massachusetts, demonstrated that marital disruption and employment status, as well as somatic symptoms and history of depression, may influence the likelihood of depressive symptoms in this population (H. Joffe, personal communication). In contrast, a community-based cross-sectional study of 2001 women in Australia showed that past and present social and physical health (including marital status, employment, report of stress and prior health problems) may influence the profile of treatment-seeking women, but appear to have little relevance for the majority of the middle-aged population.^30^ A prospective study of 2565 women (Massachusetts Women's Health Study)^31^ also confirmed that the majority of women reported relief or a neutral feeling about the event of menopause.

Higher rates of physical symptoms and depression influenced negative feelings towards the menopause, while education and marital status were not significantly correlated. In summary, poorer social and health conditions appear to negatively influence the reported experience of some but not all perimenopausal women, particularly those who report more physical and emotional symptoms and who consequently seek specific treatment for these symptoms.

## Depressive symptoms in perimenopausal women

The mechanism by which the perimenopause is associated with an increased risk for depressive symptoms in middle-aged women is still controversial.^32^ Most studies examining this question vary greatly in design. For example, data are derived from different settings (gynecological clinics versus community-based studies), and have included women with diverse menopausal status, ascribed primarily to age.^33^ These studies also suffered from a lack of standardized instruments for evaluating psychiatric symptoms.^19^ It is therefore more informative to review them separately.

### Clinic-based studies

Several investigators and previous reviews have consistently described the finding of increased reporting of severe physical and emotional complaints among women attending gynecologic clinics.^3,32^ Women who comprise these samples are not representative of the population of perimenopausal women overall but rather may represent a group with a heightened prevalence of affective disorders.^3^ In one study, Hay et al. (1994)^28^ investigated 78 peri/ postmenopausal women (mean age = 49 years) attending a "menopause clinic," and noted that 45% (N = 35) were clinically depressed, based on assessment using the Montgomery-Asberg Depression Rating Scale (MADRS). Another study describing 100 women (aged 40-60 years) attending a menopause clinic in California was conducted by Anderson et al. (1987),^27^ where 63% of patients reported emotional symptoms as the primary reason for attending the clinic. Follow-up of this cohort confirmed that 33% had moderate to severe depression based on the Zung self-rating scale scores. We recently investigated 96 endocrinologically confirmed perimenopausal women attending a gynecological clinic, and identified 46 patients (48%) suffering from significant psychiatric symptoms. Twenty-eight of these women (29.2% of the whole group) met DSM-IV criteria for Depressive Disorders (Major Depressive Disorder, Dysthymic Disorder, and Depressive Disorder Not Otherwise Specified [NOS]). There were no demographic characteristics strongly associated with depression in this population.^34^

### Cross-sectional and longitudinal studies

Most cross-sectional studies suggest that perimenopausal women (commonly defined as women aged 45-55 presenting with changes in menstrual pattern) are more likely to report depressive symptoms compared to premenopausal (i.e. women at the same age still having regular menstrual periods) or postmenopausal women.^11,19^ In one study by Ballinger (1977),^35^ in which 217 female medical outpatients (aged 40-54 years) were screened using the General Health Questionnaire, 52.5% of the sample was identified as fitting the description of "psychiatric case". Depressive symptoms in this population were more frequent among women who were perimenopausal or in those who had just become menopausal. A cross-sectional survey of 850 pre-, peri- and postmenopausal women (aged 45-65), which was representative of the general population of South-East England, demonstrated a 2530% frequency of depressed mood based on specific questions selected from the Leeds scales, which were developed to assess mood in general populations. The prevalence of depression increased significantly in peri- and postmenopausal women compared to those who were not yet menopausal.^36^ A survey of 152 women in Stockholm ^37^ identified higher prevalence of negative mood among women who reported dysmenorrhea or at least a 6-month period of amenorrhea, as well as a history of premenstrual complaints and vasomotor symptoms. Other factors, such as cigarette smoking and lack of exercise, were significantly associated with negative mood and reduced sexual interest.

The Massachusetts Women's Health Study (a 5-year prospective cohort investigation) assessed 2565 subjects (aged 45-55 years) through semi-structured interviews to obtain information regarding sociodemographic status, social supports, life style, physical health and health-care utilization, menstrual status, and depressive symptoms. Results indicated that a long period of perimenopause (as defined by experiencing a change in cycle regularity or amenorrhea for 11 months or less) was significantly associated with physical discomfort and depression (the latter assessed by the Center for Epidemiological Studies-De-pressive Scale [CES-D] scores above 15). In addition, the rate of depression decreased as subjects become postmenopausal.^31^

Kaufert et al. (1992) assessed 469 women (mean age of 48.4 years) in a 3-year prospective study (The Manitoba Project).^38^ No precise definition of the perimenopause was used. The investigators characterized as perimenopausal all women who were neither premenopausal (women reporting regular cycles) nor postmenopausal (women who reported amenorrhea at two consecutive interviews). Women who had undergone hysterectomy and oophorectomy (surgical menopause) constituted a fourth group. Over the 3-year period of the study, over 75% of the women reported some change in their menstrual status. Results indicated that depression, as measured by a CES-D score of greater than 15, was relatively persistent for 25% of women across three or more interviews. Additionally, the surgical menopause group was the only of the four groups evaluated which had any impact on the likelihood of depression (CES-D scores >15). Conversely, differences in health conditions (e.g. arthritis) and current stressors (e.g. marital problems or difficulties with children) markedly affected the likelihood of becoming depressed.

Finally, preliminary data from a prospective cohort study of older premenopausal women (aged 36-44) ("Harvard Study of Mood and Cycles");^39^ Harlow et al., 1999) suggest that reported changes in menstrual regularity are significantly associated with the new onset of depressive episodes (B. Harlow, personal communication). The extent to which reproductive endocrine markers correlate with onset or recurrence of depression among the cohort in question, as they are prospectively followed, may refine our understanding of specific populations and risk for depression during the perimenopause.

In conclusion, despite methodological limitations associated with inconsistent definitions of menopausal status and with variable criteria, most cross-sectional and prospective community-based studies suggest an association between the perimenopause and the occurrence of depressive symptoms. It remains to be more clearly delineated which circumstances may drive the observation that not all women manifest perimenopausal depressive symptoms, including psychosocial factors, hormonal changes, and past history of psychiatric illness.

### Hormonal changes and vulnerability to depression: possible models of interaction

Various theories attempt to explain the mechanism of mood changes seen in some women during the menopausal transition.^40^

Some authors suggest the existence of specific subpopulations of women with a particular vulnerability to depression during periods of intense hormonal fluctuations (reproductive- associated mood disturbance), such as premenstrual periods, the puerperium or the transition to the menopause.^41,42^ The menarcheal initiation of cyclic fluctuation of gonadal steroids (estrogen and progesterone) is coincident with the beginning of gender-based differences in rates of depression.^43-44^ It is thus intuitive that cyclic fluctuations may influence a preexisting vulnerability to mood disturbance, increasing the risk for depressive disorders. Supporting this hypothesis of a continuum of vulnerability, several studies suggest that women who suffer from severe premenstrual syndrome are more likely to present with depressive symptoms as the menopause approaches,^34,39^ and may report more physical symptoms^45^ and social dysfunction.^46^

The "estrogen withdrawal theory"^40^ proposes that onset or worsening of mood symptoms in perimenopausal women may be secondary to a hypoestrogenic state. The higher incidence of depression in women who have undergone bilateral oophorectomy (surgical menopause) compared with naturally menopausal women would strengthen this theory. Roca et al. also demonstrated a temporal correspondence between restoration of ovarian function (as measured by serum levels of FSH) and improvement in mood (based on CES-D scores) in an 8-week follow-up of perimenopausal women.^47^ Nonetheless, women with perimenopausal depressive symptoms may show diverse hormonal changes, such as lower LH and free-tryptophan levels.^48^ In addition, estrogen and progesterone levels may vary widely from one cycle to the next during the menopausal transition^2^ and may not necessarily correlate with changes in mood.

Another hormonal theory employed to explain the occurrence of depressive symptoms during the perimenopause is the "domino theory".^15,40^ This theory proposes that the discomfort caused by somatic symptoms of the perimenopause (e.g., night sweats and hot flushes) provokes physical changes (e.g. sleep disturbance) and consequently affects mood stability. Hence, investigators have speculated that the capacity of estrogen to improve mood is secondary to the relief of somatic menopausal symptoms and secondary to normalization of sleep.^49^ Schmidt et al. (1997) have recently demonstrated that estrogen replacement improves depressive symptoms in perimenopausal women who do not have hot flushes. It is possible, therefore, that the effects of estrogen on mood and on vasomotor symptoms (the latter attributed possibly to hypothalamic thermoregulatory dyscontrol) may be independent.^3^

The presence of other hormonal fluctuations at the time of the perimenopause may restrict the importance of estrogen levels per se as the critical hormonal risk factor in reproductive-endocrine-associated depressions. Results of one recent study of 50 endocrinologically confirmed perimenopausal women (as defined by history of menstrual irregularity and amenorrhea for less than 12 months, aged 4055 years, and serum levels of FSH > 25 IU/L), evaluating the antidepressant efficacy of 17 bestradiol compared with placebo, suggested that changes in serum levels of FSH (between two evaluations) correlated strongly with changes in MADRS scores, independent of the treatment applied.^50^ Despite this interesting preliminary finding, it is premature to conclude that an absolute decline in serum levels of gonadal steroids, rather than the heightened sensitivity to hormonal variability, account for the onset or exacerbation of mood disturbance in perimenopausal women.

## Depression in perimenopausal women: _○ ○_Therapeut ic implications

### General considerations

The evaluation of women aged 40-55 years who present with depressive symptoms should typically include inquiries regarding recent changes in menstrual characteristics and perimenopausal-related somatic symptoms. Such questions should complement any standard thorough psychiatric assessment.^32^ For example, questions regarding the presence of vasomotor symptoms (hot flushes or cold sweats) and patterns of sleep are crucial and should be incorporated into the clinical assessment, regardless of signs of menstrual irregularity. A decrease in sexual pleasure or desire should also be investigated, taking into account the possibility that the hormonal changes such as estrogen deficiency may result in vaginal dryness and atrophy and subsequent dyspareunia.^51^

If a woman experiences somatic or emotional symptoms but few menstrual irregularities, the investigation of serum levels of FSH and estradiol on day 2 or 3 of the cycle may be helpful. In these cases, FSH levels greater than 20 UI/L, with estradiol levels < 60 pg/ml, indicate decreased ovarian function and, combined with clinical symptoms, suggest perimenopausal status, even in the absence of irregularity in menstrual function.^32^

In patients who present with mild to moderate symptoms of depressed mood and irritability, combined with somatic symptoms of the perimenopause (including vasomotor symptoms and/or sexual dysfunction) the potential role of hormone replacement therapy (HRT) should be considered as a frontline treatment.^32^

Administration of estrogen may also help to improve quality of life and to prevent a large amount of health problems for women approaching the menopause.^52,53^ If depressive symptoms do not improve after a few weeks of hormone treatment, other interventions, including psychotherapy and antidepressant medication, should be explored. Women should also be encouraged to explore other potential sources of stress like marital relationships, shifting relationships with friends, changes in role expectations, and new-onset health problems.

Estrogen as an antidepressant agent or augmentation strategy

Several studies have investigated the putative antidepressant benefit of estrogen in different subpopulations. These are summarized in Table 1.

**Table 1:** Estrogen treatment studies of depression

	Population studied	Subjects (n)	Duration of trial	Study design	Results
**Estrogen as monotherapy**					
Campbell and Whitehead,^15^ 1977	Severe menopausal MDD	64	2 months per drug	E vs. PL 2XB, X-over	E > PL
Schneider et al.,^58^ 1977	Menopausal MDD BDI > 18	10	1 month	Open E	Significant BDI improvement
Ditkoff et al,^60^ 1991	Mixed menopausal status	36	3 months	E vs. PL, 2XB	E > PL Mood Enhancement
Schmidt et al.,^3^ 1997	Perimenopausal MDD	20	6 weeks	E vs. PL	E > PL
Gregoire et al.,^55^ 1996	Postpartum MDD	61	6 months	E + P vs. PL, 2XB	E > PL EPDS improvement
Smith et al.,^54^ 1995	Severe PMS with depressive symptoms	56	8 months	E + P	Improvement
Schneider et al.,^58^ 1977	Menopausal MDD BDI > 18	10	1 month	Open E	No significant BDI improvement
Thomson,^62^ 1976	Mixed menopausal	16	2 months	E vs. PL, 2XB	E=PL
Coope,^61^ 1991	Menopausal MDD	55	6 months per drug	E vs. PL, 2XB, X-over	Mild improvement E=PL
**Estrogen as Augmentation agent to antidepressants**					
Shapira et al.,^64^ 1985	Refractory depression	11	4 weeks	E+ imipramine	No significant improvement
Schneider et al.,^65^ 1997	Postmenopausal depressed women	367	6 weeks	ERT + FL ERT + PL Non ERT + FL Non ERT + PL	ERT increased response to FL treatment
Amsterdam et al.,^66^ 1999	Women with MDD Aged < 45 vs. ^3^45 y	568	12 weeks	Open FL Women on ERT vs. non-ERT	No additional antidepressant effect with ERT
**Estrogen** Sichel et al.,^67^ 1995	History of Puerperal Psychotic mania or severe postpartum depression	11	1 month 1 year follow-up	Open E	**Prophylaxis** All but one woman remained well (acute period or follow-up)

*MDD, major depression; BDI, Beck Depression Inventory, EDPS, Edinburgh Postnatal Depression Scale; E, estrogen-treated group; PL, placebo; ERT, estrogen replacement therapy; FL, fluoxetine; 2XB, double-blind; X-over, crossover.*

The antidepressant benefits of estrogen monotherapy for the treatment of premen-strual^54^ and postpartum^55^ depressive symptoms have been described. However, some study limitations, such as the concomitant use of antidepressants by 47% of subjects studied by Gregoire et al.^55^ may restrict the generalization of their results.

It has been shown that estrogen replacement therapy improves subjective wellbeing and quality of life during the perimenopause.^56,57^ Estrogen treatment studies also suggest superior efficacy over placebo in treating minor depression in peri- and postmenopausal women.^58-60^ However, previous studies have not consistently found that estrogen has a significant antidepressant benefit in perimenopausal women.^61,62^ More recently, a double-blind, placebo-controlled study of raloxifene (a selective estrogen receptor modulator with estrogen agonist effects) has also failed to show significant effects on mood.^63^

Previous investigation of the antidepressant effect of estrogen in peri- and postmenopausal women has been confounded by multiple factors, including the heterogeneity of methods to define and assess menopausal and hormonal status, lack of standardized diagnostic and outcome measurements, differences in hormone preparations, and the wide range of doses and different methods of administration.^43,49^

To date, only two studies^3,15^ demonstrate putative efficacy of estrogen monotherapy as a treatment for depression in peri- or postmenopausal women. We recently concluded a double-blind, placebo-controlled study demonstrating the efficacy of transdermal patches of 17 b-estradiol in the treatment of endocrinologically confirmed perimenopausal women with depression.^68^ The extent to which estrogen may be used as a primary treatment for mood disturbance, and in what population it might prove to be most efficacious, remains unclear.

## Conclusions

Review of the literature strongly suggests a relationship between the perimenopause and depressive symptoms. In addition, women who report a prior history of depression and/or experience depressive symptoms during periods of great hormonal variability (e.g. premenstrual periods and the puerperium) appear to be more vulnerable to depression at perimenopause. The extent to which fluctuations of FSH and E2 levels, rather than the absolute decline of estrogen production, may influence the recurrence or new onset of depression during the menopausal transition is still unclear. Future studies should prospectively investigate the temporal association between hormonal changes, somatic symptoms and depressive episodes, as menopause approaches.

Finally, it appears that estrogen therapy may play an important role in the treatment of depressive symptoms in this sub-population. Further clinical trials, employing long-term treatment and follow-up and evaluating the potential alteration of antidepressant benefits with concomitant progesterone (needed to reduce the risk for endometrial hyperplasia for women with an intact uterus), are necessary.
